# COVID-19 Coinfection with *Mycobacterium abscessus* in a Patient with Multiple Myeloma

**DOI:** 10.1155/2021/8840536

**Published:** 2021-01-15

**Authors:** Jose A. Rodriguez, Charles Bonnano, Pratik Khatiwada, Alejandra A. Roa, Daniel Mayer, Paula A. Eckardt

**Affiliations:** ^1^Department of Internal Medicine, Memorial Healthcare System, Pembroke Pines, FL, USA; ^2^Division of Critical Care Memorial Hospital West, Memorial Healthcare, Pembroke Pines, FL, USA; ^3^Division of Infectious Disease, Memorial Regional Hospital, Memorial Healthcare System, Hollywood, FL, USA

## Abstract

**Background:**

Coronavirus disease (COVID-19) is a worldwide pandemic causing multiple fatalities and morbidities worldwide. We report a case of severe pneumonia causing acute respiratory distress syndrome due to a coinfection with SARS-CoV-2 and *Mycobacterium abscessus* in an elderly patient with multiple myeloma in Florida, USA. *Case Presentation*. An 84-year-old male with a medical history significant for multiple myeloma not in remission was sent to the emergency department to rule out COVID-19 infection prior to continuing his chemotherapy sessions. At presentation, he had nonspecific mild symptoms and an unremarkable physical examination. He had significant blood test findings including serum lactate dehydrogenase 373 U/L, high sensitive C-reactive protein 17.40 mg/l, and ferritin 415 ng/ml. Xpert-SARS-CoV-2 was positive. Chest radiograph revealed patchy areas of interstitial infiltrates in mid to lower lung zones. During his hospitalization course, his oxygenation deteriorated, requiring mechanical intubation. Repeat chest radiograph showed worsening bilateral infiltrates. He was started on broad-spectrum antibiotics and eventually weaned off mechanical intubation and extubated. On the 11^th^ day of admission, he was found to be bradycardic and in shock, and he was reintubated. His labs showed worsening inflammatory markers along with kidney dysfunction to the point of requiring renal replacement therapy. He received both convalescent plasma and remdesivir for treatment of COVID-19 pneumonia. Eventually, repeat blood cultures came back positive for the growth of acid-fast beaded bacilli. While awaiting final culture and sensitivity reports, his antibiotics were upgraded to cover possible nocardia infection. Repeat blood and sputum cultures resulted in growth of AFB bacilli *Mycobacterium abscessus* 1 week after.

**Conclusions:**

This case report highlights the importance of keeping a broad differential and considering multiple coinfections, including atypical ones during this COVID-19 pandemic, such as the one that was discussed above, *Mycobacterium abscessus*, in order to provide goal-directed therapy.

## 1. Background

COVID-19 is a novel coronavirus originating out of Wuhan City, Hubei Province, China. Coronaviruses are a large family of viruses causing respiratory diseases ranging from less severe such as common cold to more severe diseases such as Middle East respiratory syndrome (MERS) and Severe Acute Respiratory Syndrome (SARS) [1].

*Mycobacterium abscessus*, a nontuberculosis mycobacteria (NTM), comprises three subspecies: *M. abscessus* subsp. *abscessus*, *M. abscessus* subsp. *bolletii*, and *M. abscessus* subsp. *massiliense*. *Mycobacterium abscessus* is a rapidly growing mycobacterium (RGM) that is found in the environment that can cause skin, soft tissue, pulmonary, and bone infection [2].

There have been multiple cases reporting coinfection of COVID-19 with other viruses such as influenza A, parainfluenza, and respiratory syncytial virus [3–5] and cases of COVID-19 with associated pulmonary tuberculosis [6]. However, through our searching, we have not yet come across a case involving COVID-19 pneumonia with coinfection of *Mycobacterium abscessus*.

## 2. Case Presentation

An 84-year-old male with a medical history significant for multiple myeloma not in remission, insulin-dependent type II diabetes mellitus, hypertension, and hyperlipidemia presented to our emergency department to be tested for COVID-19 infection. He was diagnosed with multiple myeloma about 6 months ago at another institute and was undergoing cycles of outpatient chemotherapy with Revlimid, Velcade, and Dexamethasone (RVD; 1.3 mg/m^2^ SC). He was sent to the ED to rule out COVID-19 infection prior to him continuing his chemotherapy sessions. At that time, he was experiencing nonspecific symptoms such as malaise, fatigue, hyporexia, and aguesia for the past 7 days but denied fever, chest pain, cough, difficulty in breathing, rhinorrhea, nasal congestion, sore throat, sneezing, or any other URI symptoms at the time of presentation. He was in close contact with his daughter who was diagnosed with COVID-19 infection. Home medications included basal bolus of insulin, acarbose, liraglutide, and linagliptin for his diabetes and carvedilol, lisinopril, furosemide, and verapamil for his hypertension and was on high-intensity statin therapy. He was a former smoker who quit about 35 years ago. He reported no use of alcohol or illicit drugs. He was followed up by an oncologist part of our healthcare system.

**Figure 1:**
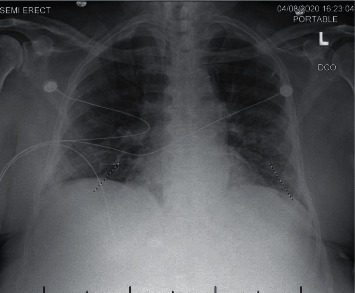
Portable chest X-ray showing bilateral interstitial infiltrates on admission.

On physical examination at the time of presentation, the patient was afebrile (36°C), with a blood pressure of 156/64 mmHg, heart rate of 94 beats/min, respiratory rate of 19 breaths/min, and oxygen saturation of 95% on room air. He was not in acute distress, and chest auscultation revealed normal breath sounds. Rest of the physical examination was unremarkable. Initial blood tests revealed the following: hemoglobin level 11.3 g/dL, leucocyte count 3,600 cells/mm^3^, with 74% neutrophils, 17.6% lymphocytes, and 6.4% monocytes, platelet count 106,000/mm^3^, serum sodium level 135 mmol/L, urea 40 mg/dL, creatinine level 2.42 mg/dL, lactate dehydrogenase 373 U/L, high sensitive C-reactive protein 17.40 mg/l, and ferritin level 415 ng/ml. Xpert-SARS-CoV-2 obtained through a nasopharyngeal swab was positive. Chest radiograph revealed patchy areas of interstitial infiltrates in mid to lower lung zones (Figure 1).

The patient was admitted to telemetry floors with the diagnosis of mild COVID-19 pneumonia and acute kidney injury (AKI). He was started on hydroxychloroquine and azithromycin for COVID-19 pneumonia, supplemental oxygen as needed to maintain an oxygen saturation >92%, Lovenox for thromboembolism prophylaxis, supportive measures with intravenous fluids, and electrolyte abnormalities correction for his AKI. Except for spikes in temperature and mild hypoxia requiring 4 L of O_2_ through a nasal cannula, his hospital course was unremarkable for the first 5 days.

On the sixth day of admission, the patient was found to have hematochezia associated with a significant drop in his hemoglobin from 10 mg/dL to 7.1 mg/dL. He had a history of diverticulosis from previous colonoscopy. He was managed conservatively with holding antiplatelet and anticoagulation and started on intravenous proton-pump inhibitors. After the acute blood loss anemia, the patient became more hypoxic with an increment in supplemental oxygen requirements. He underwent emergent intubation and mechanical ventilation in the setting of acute hypoxemic respiratory failure and was transferred to an intensive care unit. He was also resuscitated with intravenous blood products. Repeat chest radiograph was remarkable for worsening bilateral infiltrates, and the patient was started on empiric treatment for superimposed bacterial infection with cefepime 2 g IV and vancomycin. At that time, his COVID-19 infection was reassessed as severe multifocal COVID-19 pneumonia. IL-6 level and hepatitis B and C were ordered for possible consideration of tocilizumab therapy. Convalescent plasma and remdesivir trials were also considered for treatment therapy. His IL-6 level was elevated at 38.73 pg/mL and also found to have hepatitis B surface antigen, core antibody. It was decided to abstain from tocilizumab therapy given the possibility of reactivation of his chronic hepatitis B. He was able to be weaned off the mechanical ventilation and was switched to nonrebreather after 5 days of intubation and mechanical ventilation.

On the 11^th^ day of admission, the patient was found to be bradycardic and in shock and eventually found to be in asystole prompting ACLS with return of circulation after 7 minutes and 2 rounds of epinephrine. The patient developed cardiac arrest and had to be reintubated and mechanically ventilated. His labs showed worsening inflammatory markers, as well as kidney function, requiring renal replacement therapy. He received both convalescent plasma and remdesivir 200 mg IV BID loading dose followed by 100 mg IV BID daily for 4 days for treatment of COVID-19 pneumonia.

**Figure 2:**
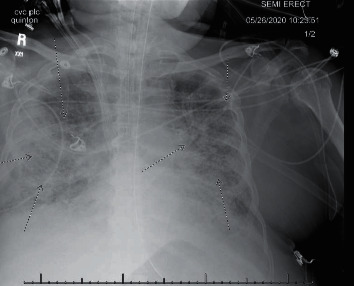
Portable chest X-ray with lungs demonstrating extensive diffuse infiltrates with worsening aeration from the prior chest X-ray.

He was kept on mechanical ventilation with a low-stretch tidal volume strategy, 6 ml/kg TV, goal plateau pressure less than 30, permissive hypercapnia with pH >7.20, and high PEEP with the goal for lower FiO_2_ to prevent hyperoxia; however, he continued to have significant shunt hypoxemia due to volume overload and severe pneumonitis. In the following days, he remained on mechanical ventilation and hemodialysis without any significant improvement in the ventilator settings. In the context of shock, requiring vasopressors such as norepinephrine, increased FiO_2_, bandemia (18%), and positive procalcitonin level, repeat cultures were sent. CXR revealed worsening infiltrates (Figure 2). New-onset septic shock with possible ventilator-associated pneumonia was considered. Repeat blood and sputum cultures came back positive for the growth of acid-fast beaded bacilli. With uncertainty, if the results were contamination versus an isolated line infection, repeat AFB blood cultures from 2 sites including the lines were sent along with AFB sputum culture for confirmation. While awaiting final culture and sensitivity reports, his antibiotics were upgraded to cover possible nocardia infection with imipenem/cilastatin 500 mg IV and trimethoprim/sulfamethoxazole IV 5 mg/kg. Unfortunately, he was unable to withstand multiorgan failure with septic shock and ARDS complicated by AFB bacteremia. He developed asystole with fixed and dilated pupils and was pronounced dead after approximately 2 months being hospitalized. Blood and sputum cultures 1 week after came back positive for AFB bacilli *Mycobacterium abscessus*.

## 3. Discussion

COVID-19 infection is primarily transmitted via respiratory droplets and contact routes [7]. Clinical features include fever, shortness of breath, fatigue, loss of appetite, sore throat, nausea, and vomiting, with the development of bilateral pneumonia [8]. Proper testing techniques are crucial as inappropriate methods can lead to false-negative results. Initial diagnostic testing includes samples from the upper respiratory specimen by either nasopharyngeal swab, oropharyngeal swab, or sputum collection (not inducible). Patients who are intubated can have samples collected from respiratory tract aspirate or bronchoalveolar lavage [9].

Predisposing conditions that are commonly associated with *M. abscessus* pulmonary infections are bronchiectasis with reticulonodular lung infiltration, cystic fibrosis, and lipoid pneumonia. It is the third most frequently isolated respiratory NTM and gives more than 80% of all RGM respiratory infections [2, 10]. The current 2020 guideline for treatment of NTM recommends using clinical, radiographic (nodular or cavitary opacities on the chest radiograph or a tomography scan showing bronchiectasis with multiple small nodules), and microbiologic (positive cultures from at least 2 separate expectorated sputum samples, or 1 culture from bronchial lavage or biopsy with positive culture) criteria for diagnosing NTM pulmonary disease [10].

The clinical presentation of these two diseases has very similar traits. A retrospective case series involving 393 patients with confirmed COVID-19 had consistent characteristics, with cough being the most prevalent (79.4%) and fever, dyspnea, myalgia, diarrhea, and nausea and vomiting; most patients had lymphopenia (90%) and elevated inflammatory markers (ferritin, CRP, D-dimer, and procalcitonin) [8]. In a retrospective analysis of 154 patients with pulmonary disease due to RGM in which 80 percent of the cases had *M*. *abscessus*, findings were consistent, with cough being the most prevalent symptom and fever, weight loss, and hemoptysis; equally common chest radiograph patterns were interstitial, mixed interstitial with alveolar infiltrates, and reticonodular. Death was attributed directly to progressive RGM lung disease with respiratory failure in 14% of the cases [11]. As we can see by comparing these retrospective studies, there is significant cross over in regard to symptomology, especially in the immunocompromised patient, such as ours.

Coinfection with COVID-19 has been a major concern for the healthcare system. Recent studies from JAMA have shown that approximately 20% of COVID-19 patients were positive with one or more additional viral pathogens, the most common being rhinovirus, respiratory syncytial virus, and non-SARS-CoV-2 coronaviridae [12]. Searches have been underway for coinfection of COVID-19 with superimposed bacterial/fungal infections; however, data are scarce. An article was published by the Oxford University Press for the Infectious Disease Society of America where they conducted a search for data regarding bacterial/fungal coinfections, treatments, and outcomes. Their search resulted in 1007 abstracts, in which they concluded, for COVID-19, 8% of patients were reported as experiencing bacterial/fungal coinfection during hospital admission [13].

Treatment plans differentiate significantly compared to COVID-19 and NTM even though their symptomology might be similar. Unfortunately with COVID-19, clinical trials and investigational therapies continue for more optimal outcomes. Due to this sudden pandemic, all of the recommended medications are FDA approved under an Emergency Investigational New Drug Application. The most recent recommendations include remdesivir and convalescent plasma. Remdesivir (200 mg loading dose on day 1, followed by 100 mg daily for up to 9 additional days) was shown to shorten the recovery time in adults hospitalized with COVID-19 [13]. Studies for convalescent plasma showed potential of stopping viral shedding and extending survival in patients with COVID-19 and respiratory failure. Timing of initiation of this therapy was crucial as patients with end-stage disease did not have any benefit [14]. Treatments for RGM differ based on clinical presentation. In this situation, we will focus on treatment for pulmonary disease. The optimal drugs, regimens, and duration of therapy are unknown and require further research. However, given the difficulty of treatment and time it takes for sensitivities to result, starting patients on broad empiric antibiotics is common. Sensitivities direct antimicrobial therapy, but even then, eradication is usually very hard to achieve. Initiation of therapy typically includes IV imipenem 500–1,000 mg two to three times per day or cefoxitin 2–4 gm two to three times per day and amikacin 10–15 mg/kg IV per day plus a macrolide (e.g., azithromycin 250–500 mg per day) for 8–12 weeks with conversion to oral agents plus or minus a macrolide, based on sensitivities, for the remaining 12 months [10]. Disseminated and pulmonary disease are notoriously refractory to therapy, and outcomes are poor with half or more of patients remaining culture positive or relapsing, particularly with infection due to strains with inducible macrolide resistance such as *M. abscessus* subspecies *abscessus* [15]. If antimicrobial therapy is unsuccessful, surgical intervention is an alternative approach. Studies have shown that surgery was estimated as acceptable and feasible as an adjunct to medical therapy [10].

The importance of this case was to discuss and inform our readers of the importance of maintaining an open mind frame of differentials even during a pandemic. We presented an elderly gentleman immunocompromised from recent diagnosis of MM on chemotherapy who presented initially with a relatively mild COVID-19 infection. During hospitalization for any patient, once their clinical picture has changed, full investigation should be sought to identify any causative agent. In this scenario, on day 11, once his respiratory status declined further, collecting repeat cultures may have been useful. Nonetheless, he was on broad-spectrum antibiotics for any possible concerning coinfection. At the time cultures were positive for AFB, his status was so poor that adjusting medical therapy would not have resulted in a different outcome.

## 4. Conclusions

Even when patients seem to be primarily infected with COVID-19 and are critically ill, it is important to assess for other differentials such as superimposed infections especially in the immunocompromised population, as proper antimicrobial therapy can alter outcomes.

## Data Availability

Data used to support the findings of this study are available on request.
